# A teach approach: visual mapping of chromosome translocation

**DOI:** 10.3402/meo.v21.30816

**Published:** 2016-02-02

**Authors:** Pengcheng Han

**Affiliations:** Dignity Health St Joseph’s Hospital and Medical Center, Phoenix, AZ, USA

Visual illustration is a common tool in presentation and public speech as it helps to clarify complex relations and leave long-lasting memory. Visual mapping is a method to mark the semantic content in a graphic representation. For example, it is a mental challenge to memorize a list of eight cities: Boston (MA), Charleston (SC), Los Angeles (CA), Miami (FL), New Orleans (LA), San Francisco (CA), Seattle (WA), and St Louis (MO). When this list is marked on a map, it becomes apparent that three cities are located along the west coast from north to south (Seattle, San Francisco, Los Angeles); three cities are located along the east coast from north to south (Boston, Charleston, Miami); and two cities are in the mid-country along the Mississippi river (St Louis, New Orleans). People feel it easier to memorize the map than the semantic list.

**Fig. 1:**
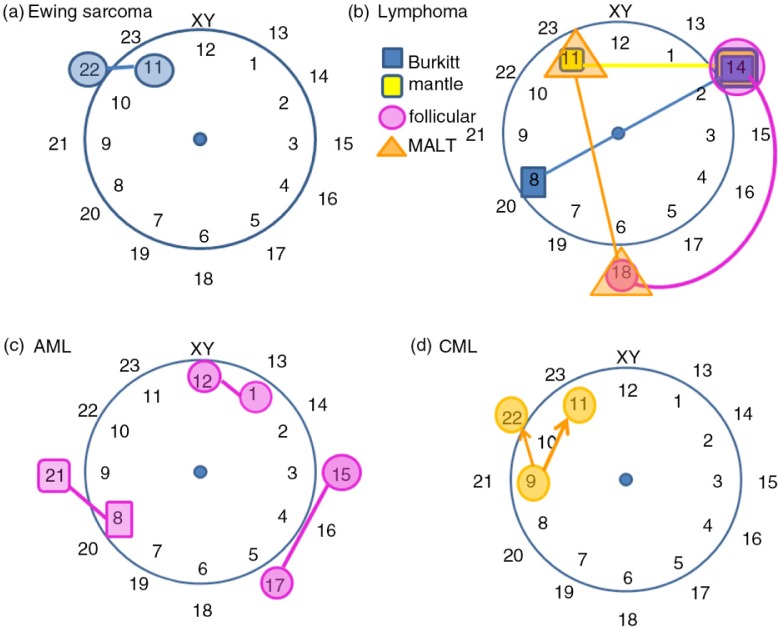
Chromosome clock model (CCM) and the visual mapping of chromosome translocation. The chromosome translocation was presented according to diseases: Ewing Sarcoma (a), lymphoma (b), acute myelogenous lymphoma (c), and chronic myelogenous lymphoma (d).

Visual mapping is a useful tool to illustrate medical knowledge points and help the learners memorize them more efficiently. In my teaching experience, I found that many learners feel hard to memorize the chromosome translation (1) (Table 1). Therefore, I designed a chromosome clock model to map these translocations (Fig. 1). Human chromosome number (23+XY) is perfect to be mapped on a clock dial in a 24-h format: the inner circle represents time in AM and the outer circle represents PM (Fig. 1). Then, we map the chromosome translocations listed in Table 1, according to the disease categories. I use the following description as a hint to help learning and memory.

**Table 1:** Ten commonly tested chromosome translocations

Chromosome Translocation	Disease
-t(1;12)	AML
-t(8;14)	Burkitt lymphoma
-t(8;21)	AML
-t(9;11)	CML
-t(9;22)	CML
-t(11;14)	Mantle cell lymphoma
-t(11;18)	MALToma
-t(11;22)	Ewing sarcoma
-t(14;18)	Follicular lymphoma
-t(15;17)	AML-M3

AML, acute myelogenous leukemia; CML, chronic myelogenous leukemia.

*Ewing sarcoma* [t(11;22)]: a localized tumor marked as a short line at 11 o'clock.

*Lymphoma*. The hubs (11, 14, 18) make a slice of apple pie, and a knife [t(8;14)] cuts through this slice of pie. Follicular lymphoma is the only round curve connecting 14 and 18 because ‘follicle’ means round. Mantle cell lymphoma is the top line [t(11;18)] because ‘mantle’ means cover on top. Burkitt lymphoma [t(8,14)] is usually a young-kid disease; Young kids like to cut and eat pie.

Acute myelogenous leukemia (AML). Two short lines are placed in parallel: t(8;21) and t (1;12). M3 is a special type of AML amenable to retinoid treatment. Because it is special, the line is not parallel but orthogonal [t(15;17)]. In addition, the three points are the time we start to work (8 o'clock), have lunch (12 o'clock), and have the afternoon tea break (15 o'clock).

Chronic myelogenous leukemia (CML). The famous Philadelphia chromosome is t(9;22). Northeast from Philly, New York City is sadly associated with 9/11.

**Fig. 2:**
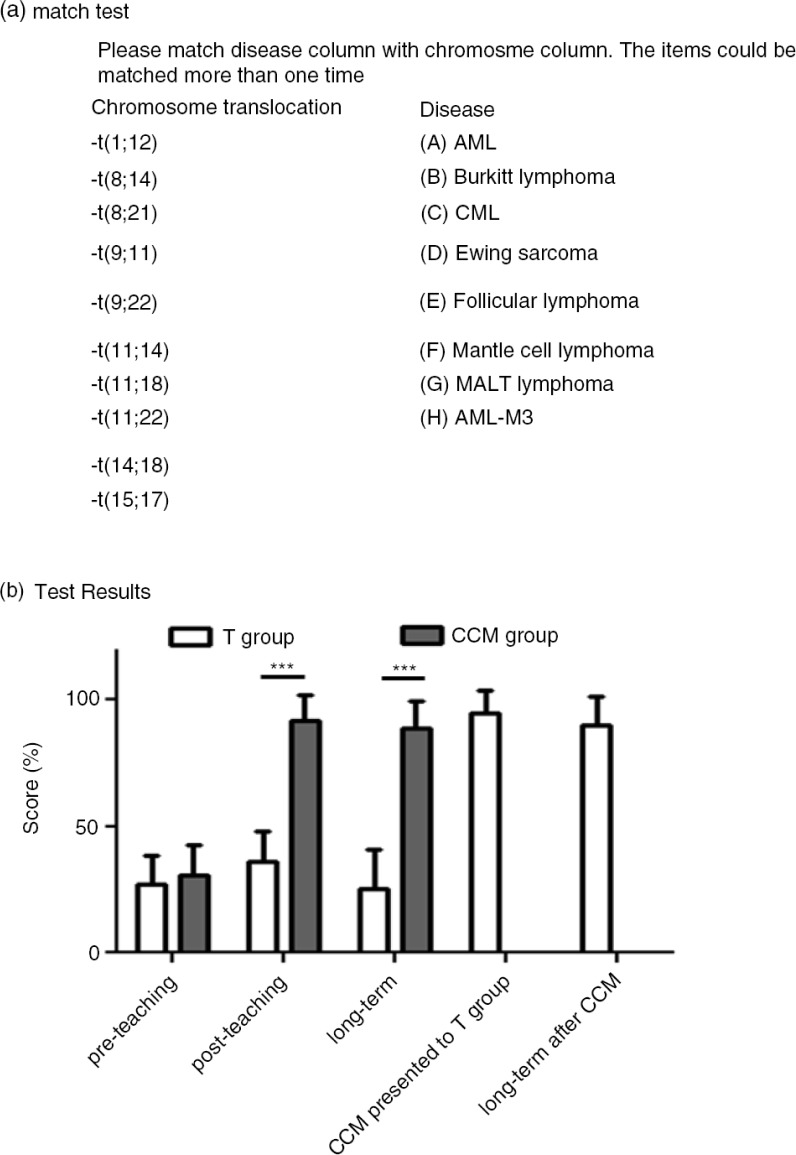
(a) The match test questionnaire used to calculate the correct percentage score (%). (b) Test and re-test for the group trained with chromosome clock model (CCM group) and for the group trained with Table 1 presentation only. After completing the experiment, the T group was remedially presented with CCM. Note that the T group performance improved after CCM compared with the initial CCM group. *** indicates *p*<0.001 (student *t*-test).

We tested the chromosome clock model (CCM) teaching approach in a total of 33 students. The students were randomized to either CCM teaching group (*n*=15) or a control group presented only with Table 1 (T group, *n*=18). We used a 10-pair match test questionnaire to examine the knowledge retained (Fig. 2a) and the percentage of corrected answers was defined as the test score. Before formal teaching, all groups were presented with Table 1 for 10 min, and the students were instructed to memorize to the best of their ability. Then, we gave a pre-teaching test and the two groups performed to the equal level (Fig. 2b). Subsequently, the CCM group was presented with Fig. 1 along with the verbal hint, as described above. The students were encouraged to practice drawing Fig. 1. The T group was presented with Table 1, a second time. Then we did post-teaching test immediately after the teaching session. Two weeks after this teaching, we re-tested these two groups without prior notice (long-term test). The CCM group performed significantly better in the post-teaching test and long-term test (student *t*-test). In the CCM group, we saw a remarkable improvement in post-teaching test compared with pre-teaching test, and much of the learned materials were successfully retained in the long-term test (Fig. 2b). At the conclusion of this experiment, we offered CCM teaching to the T group, which showed improved memory as well (Fig. 2b).

In conclusion, we provide a novel teaching approach to help learners better memorize the most commonly asked chromosome translocation. We have tested the effectiveness of this approach and hope it benefits all interested readers.

*Pengcheng Han* Dignity Health St Joseph's Hospital and Medical Center Phoenix, AZ, USA Pengcheng.Han@dignityhealth.org
